# MiR-26a/miR-26b represses tongue squamous cell carcinoma progression by targeting PAK1

**DOI:** 10.1186/s12935-020-1166-6

**Published:** 2020-03-14

**Authors:** Zhenxing Wei, Kunpeng Chang, Chongsheng Fan, Yang Zhang

**Affiliations:** Department of Otorhinolaryngology-Head and Neck Surgery, The Luoyang Central Hospital Affiliated to Zhengzhou University, No. 288 Middle Zhongzhou Road, Xigong District, Luoyang, 471000 Henan China

**Keywords:** TSCC, microRNA-26a, microRNA-26b, PAK1, Glycolysis

## Abstract

**Background:**

Tongue squamous cell carcinoma (TSCC) is the most common oral malignancy. Previous studies found that microRNA (miR)-26a and miR-26b were downregulated in TSCC tissues. The current study was designed to explore the effects of miR-26a/miR-26b on TSCC progression and the potential mechanism.

**Methods:**

Expression of miR-26a, miR-26b and p21 Activated Kinase 1 (PAK1) in TSCC tissues and cell lines was detected by reverse transcription- quantitative polymerase chain reaction (RT-qPCR). Flow cytometry analysis was performed to examine cell cycle and apoptosis. Transwell assay was conducted to evaluate the migrated and invasive abilities of SCC4 and Cal27 cells. In addition, western blot assay was employed to analyze the protein level. Glucose assay kit and lactate assay kit were utilized to analyze glycolysis. Dual-luciferase reporter and RNA immunoprecipitation (RIP) assays were applied to explore the relationship between miR-26a/miR-26b and PAK1. Xenograft tumor model was constructed to explore the role of miR-26a/miR-26b in vivo.

**Results:**

Both miR-26a and miR-26b were underexpressed, while PAK1 was highly enriched in TSCC. Overexpression of miR-26a and miR-26b inhibited TSCC cell cycle, migration invasion and glycolysis, while promoted cell apoptosis. Both miR-26a and miR-26b directly targeted and negatively regulated PAK1 expression. Introduction of PAK1 partially reversed miR-26a/miR-26b upregulation-mediated cellular behaviors in TSCC cells. Gain of miR-26a/miR-26b blocked TSCC tumor growth in vivo.

**Conclusion:**

MiR-26a/miR-26b repressed TSCC progression via targeting PAK1 in vitro and in vivo, which enriched our understanding about TSCC development and provided new insights into the its treatment.

## Highlights

1. MiR-26a and miR-26b are downregulated.
2. Upregulation of miR-26a/miR-26b represses cell cycle, migration, invasion and glycolysis, while promotes cell apoptosis of TSCC cells in vitro.
3. PAK1 is upregulated in TSCC.
4. Both miR-26a and miR-26b target PAK1.
5. MiR-26a/miR-26b inhibits TSCC progression by targeting PAK1.
6. Introduction of miR-26a/miR-26b blocks TSCC tumor growth in vivo.

## Background

TSCC is the most familiar form of oral cancer, with quick progress plus easy metastasis, which usually causing trouble of chew, speech and ingurgitation [1, 2]. There were approximately 48,100 new cases and 22,100 deaths related to TSCC in China in 2015 [3]. Despite treatment options for TSCC got greatly improved, like surgical excision, chemotherapy and radiotherapy, the treatment outcome of patients with TSCC was still very poor [4]. Therefore, it is necessary to explore mechanisms involved in the progression of TSCC so as to develop new therapeutic approaches for TSCC patients.

MicroRNAs (miRNAs) are highly conserved ncRNAs with approximately 22 nucleotides that can play vital regulatory roles in animals and plants by targeting message RNAs (mRNAs) for cleavage or translational inhibition [5]. It was determined that certain miRNAs participated in cellular behaviors, such as cell-cycle regulation, inflammation, stress response, migration, invasion, differentiation and apoptosis [6]. Dysregulation of miRNAs is commonly observed in diverse tumors, including TSCC, and closely related to cancer progression, functioning as either tumor oncogenes or suppressors [7, 8]. MiR-26a and miR-26b make up the miR-26 family [9]. MiR-26a and miR-26b are commonly dysregulated in various cancers, as well as involve in multiple biological processes via targeting corresponding mRNAs [10, 11]. Previous studies indicated that the expression of miR-26a and miR-26b was downregulated in tongue squamous cell carcinoma [11, 12]. However, the definite molecular mechanism of miR-26a/miR-26b related to TSCC progression needs further investigation.

PAKs are a group of serine/threonine kinases that function as downstream nodes for kinds of oncogenic signaling pathways; among which PAK1 locates at 11p13 region [13]. Additionally, PAKs have been involved in certain pathological conditions, including cancer [14]. Upregulation of PAK1 was reported in diverse human cancers, such like thyroid cancer [15], ovarian cancer [16], bladder cancer [17] and TSCC [18]. Through bioinformatic analysis, we observed that PAK1 could bind with miR-26a/miR-26b. Nevertheless, the potential mechanism of PAK1 in miR-26a/miR-26b-mediated TSCC calls for deeper investigation.

In our study, we investigated the influence of miR-26a/miR-26b on cell cycle, migration, invasion, glycolysis and apoptosis of TSCC in vitro and on tumor propagation in vivo, as well as explored underlying molecular mechanism.

## Materials and methods

### Patients and tumor tissues

44 paired TSCC tumor tissues and matched normal tissues were collected from patients with TSCC at the Luoyang Central Hospital Affiliated to Zhengzhou University, which were diagnosed and received tumor resection surgery between 2016 and 2018. All patients did not undergo radiotherapy, chemotherapy or other targeted therapy before surgery. All tissues were immediately put into liquid nitrogen after collection and kept at − 80 °C. This study got approval of the Ethics Committee of the Luoyang Central Hospital Affiliated to Zhengzhou University and each patient offered the written informed consent.

### Cell culture and transient transfection

Cal27, SCC4 and SCC9 cells as well as 293T cells were obtained from ATCC (Manassas, VA, USA). UM1 cells were purchased from Japanese Collection of Research Bioresources (JCRB) cell bank (Osaka, Japan), and normal human oral keratinocyte (NHOK) was supplied by ScienCell Research Laboratories (San Diego, CA, USA). Above cells were cultured in Dulbecco’s Modified Eagle Medium (DMEM, PAN Biotech, Aidenbach, Germany) added with fetal bovine serum (FBS; 10%, HyClone, Logan, UT, USA) at 37 °C with 5% CO_2_.

Mimic of miR-26a (miR-26a) or miR-26b (miR-26b) and the mimic negative control (miR-NC), inhibitors of miR-26a (anti-miR-26a) or miR-26b (anti-miR-26b) and the inhibitor negative control (anti-miR-NC) and pcDNA-PAK1 (PAK1) were acquired from GenePharma Co., ltd. (Shanghai, China). And the pcDNA empty vector (pcDNA) was purchased from Thermo Fisher Scientific (Waltham, MA, USA). SCC4 and Cal27 cells were sowed into 6-well plates (about 2 × 10^6^ cells/well) and transfected with aforementioned nucleotides or plasmids when confluence reached 70–80% using Lipofectamine 2000 (Life Technologies Corporation, Carlsbad, CA, USA) referring to manufacturer’s recommendations.

### RT-qPCR

The total RNA was isolated from tissues or transfected cells with miRNeasy Mini Kit (QIAGEN, Hilden, Germany). As for the detection of miR-26a and miR-26b enrichment, TaqMan reverse transcription kit and TaqMan MicroRNA Assays (Thermo Fisher Scientific) were employed to conduct reverse-transcription and qPCR, and U6 served as an internal control. For PAK1 abundance detection, total RNA was extracted using TRIzol^®^ reagent (Solarbio, Beijing, China). And complementary DNA was generated using M-MLV Reverse Transcriptase (Solarbio). 2× SYBR Green PCR Mastermix (Solarbio) was used to evaluate the mRNA level of PAK1, with Glyceraldehyde-3-phosphate dehydrogenase (GAPDH) as an internal reference. The relative expression levels were assessed using the 2^−∆∆Ct^ method. Primers sequences used were listed as below: miR-26a-forward (F), 5′-CTCAACTGGTGTCGTGGAGTCGGCAATTCAGTTGAGAGCCTATC-3′, miR-26a-reverse (R), 5′-ACACTCCAGCTGGGTTCAAGTAATCCAGGATA-3′; miR-26b-F, 5′-CCGGGACCCAGTTCAAGTAA-3′, miR-26b-R, 5′-CCCCGAGCCAAGTAATGGAG-3′; U6-F, 5′-CTCGCTTCGGCAGCACA-3′, U6-R, 5′-AACGCTTCACGAATTTGCGT-3′; PAK1-F, 5′-GGTGGTGGCTGCACAGTAG-3′, PAK1-R, 5′- TCTGAGGCAGGAGGTGGTAA-3′; GAPDH-F, 5′-AATCCCATCACCATCTTCC-3′, GAPDH-R, 5′-CATCACGCCACAGTTTCC-3′.

### Cell cycle assay

Transfected SCC4 and Cal27 cells were gathered, washed with pre-cold PBS and fixed with 70% ethanol. Following overnight incubation, cells were washed and stained with propidium iodide reagent in the dark. The distribution of cells in G0-G1, S and G2-M phases was determined by a flow cytometer (Countstar, Shanghai, China).

### Transwell assay

To measure migration, transfected SCC4 and Cal27 cells (1 × 10^5^) were seeded in DMEM in the higher portion of each chamber (8 µm; Corning Inc., Corning, NY, USA), while DMEM with 10% FBS was put into the lower portion of each chamber. After 48 h, cells sticking to the base of the higher portion were stained with 0.1% crystal violet, then photographed and counted using X-71 inverted light microscope (Olympus, Tokyo, Japan). Invasion assay was implemented using the similar protocols but instead, the upper compartment was coated with Matrigel (Corning Inc.) and dried overnight under sterile condition.

### Apoptosis analysis

Transfected SCC4 and Cal27 cells were gathered, washed twice with pre-cold PBS and resuspended in 200 µL binding buffer. Then Annexin V-FITC Apoptosis Detection Kit (Beyotime, Shanghai, China) was applied to evaluate apoptotic cells according to the manufacturer’s instructions. Apoptotic rates were determined utilizing a flow cytometer (Countstar) based on the analysis of the proportion of cells at Annexin V+/PI±.

### Western blot analysis

Western blot analysis was applied to analyze the protein levels of matrix metalloproteinase 9 (MMP9, cell migration marker), Cyclin D1 (CCND1, cell cycle marker), Proliferating Cell Nuclear Antigen (PCNA, cell proliferation marker), B-cell lymphoma-2 (Bcl-2, cell apoptosis marker), Bcl-2-Associated X (Bax, cell apoptosis marker) and PAK1. Firstly, total protein was isolated from SCC4 and Cal27 cells using Radio-Immunoprecipitation Assay buffer (Beyotime). Then extracted samples (30 µg/lane) were loaded onto 12% sodium dodecyl sulphate-polyacrylamide gel electrophoresis (SDS-PAGE) gels and then transferred onto polyvinylidene difluoride membrane (Bio-Rad Laboratories, Inc., Hercules, CA, USA). The membranes were saturated with 5% non-fat milk for 2 h, and incubated with specific antibodies against MMP9 (1:1500 dilution, HPA001238), CCND1 (1:1000 dilution, HPA027802), PCNA (1:1500, SAB4502103), Bax (1:1000 dilution, SAB4502546), Bcl-2 (1:1000 dilution, SAB4500003), PAK1 (1:1000 dilution, SAB4502065) or anti-β-actin (1:3000 dilution, SAB5500001) at 4 °C overnight, respectively; followed by incubation with corresponding secondary antibody (1:10,000 dilution, 12-348) for 2 h. Protein blots were motivated using ECL western blot detection system (Millipore, Billerica, MA, USA), with β-actin as a control. Above antibodies were acquired from Sigma (Saint Louis, Missouri, USA). All uncropped immunoblot images with molecular weight markers were displayed in Additional files 1, 2, 3, 4.

### Glucose consumption and lactate production analysis

Glycolysis could be assessed by glucose consumption and lactate production. At 48 h post transfection, the medium, SCC4 and Cal27 cells were gathered, and the content of glucose and lactate in medium was analyzed by the usage of the glucose assay kit (Biovision, Milpitas, CA, USA) and lactate assay kit (Biovision) in conformity to the supplier’s instructions.

### Bioinformatic analysis

The prediction of potential target mRNAs of miR-26a/miR-26b was conducted using the convincing online prediction website DIANA TOOL (http://carolina.imis.athena-innovation.gr/diana_tools/web/).

### Dual-luciferase reporter assay

Then the 3′UTR segments of PAK1 harboring the binding sites for miR-26a and miR-26b (the same two) were amplified by PCR and subcloned into the pGL3 luciferase promoter vector (pGL3-empty, Promega, Madison, WI, USA), signed as PAK1-WT1 and PAK1-WT2, respectively. In addition, their mutants obtained by mutating the binding sites were also amplified and subcloned into the vector to construct corresponding PAK1-MUT1 and PAK1-MUT2, respectively. Then, 293T cells were co-transfected with PAK1-WT or PAK1-MUT, along with miR-26a, miR-26b or miR-NC utilizing Lipofectamine 2000 (Life Technologies Corporation). After 48 h, the luciferase activities were assessed via Dual-Luciferase Reporter System (Beyotime) following the protocols of manufacturer.

### RIP assay

The commercial EZ-Magna RIP Kit (Millipore) was applied for the RIP assay. SCC4 and Cal27 cells were harvested when confluence reached 80% and then lysed using pre-coat RIP lysis buffer. The extract was then incubated with anti-Ago2 or anti-IgG (Sigma). After overnight incubation, proteinase K was used to digest the protein in the samples, and the immunoprecipitated RNA was isolated, purified and subjected to RT-qPCR assay.

### Xenograft tumor assay

The experiment in nude mice was permitted by the Ethics Committee of the Luoyang Central Hospital Affiliated to Zhengzhou University. 20 male BALB/c nude mice (4–6-week-old) were purchased from Beijing Laboratory Animal Center (Beijing, China) and evenly divided into four groups (n = 5). SCC4 cells stably introduced with nothing (Empty group), miR-NC (miR-NC group), miR-26a (miR-26a group) and miR-26b (miR-26a group) were injected into the right flank of nude mice. The volume of formed tumors was monitored once a week using the formula, Volume = length × width^2^/2. 4 weeks later, all mice were killed to resect the tumors, then tumors were weighed.

### Statistical analysis

All data were derived from at least 3 repetitions and shown as mean ± standard deviation and analyzed utilizing SPSS 21.0 software (SPSS, Chicago, IL, USA). The comparison was executed using Student’s *t*-test or one-way analysis of variance followed by Tukey test. *P* less than 0.05 was recognized as statistically significant.

## Results

### Both miR-26a and miR-26b were downregulated in TSCC tissues and cell lines

**Fig. 1:**
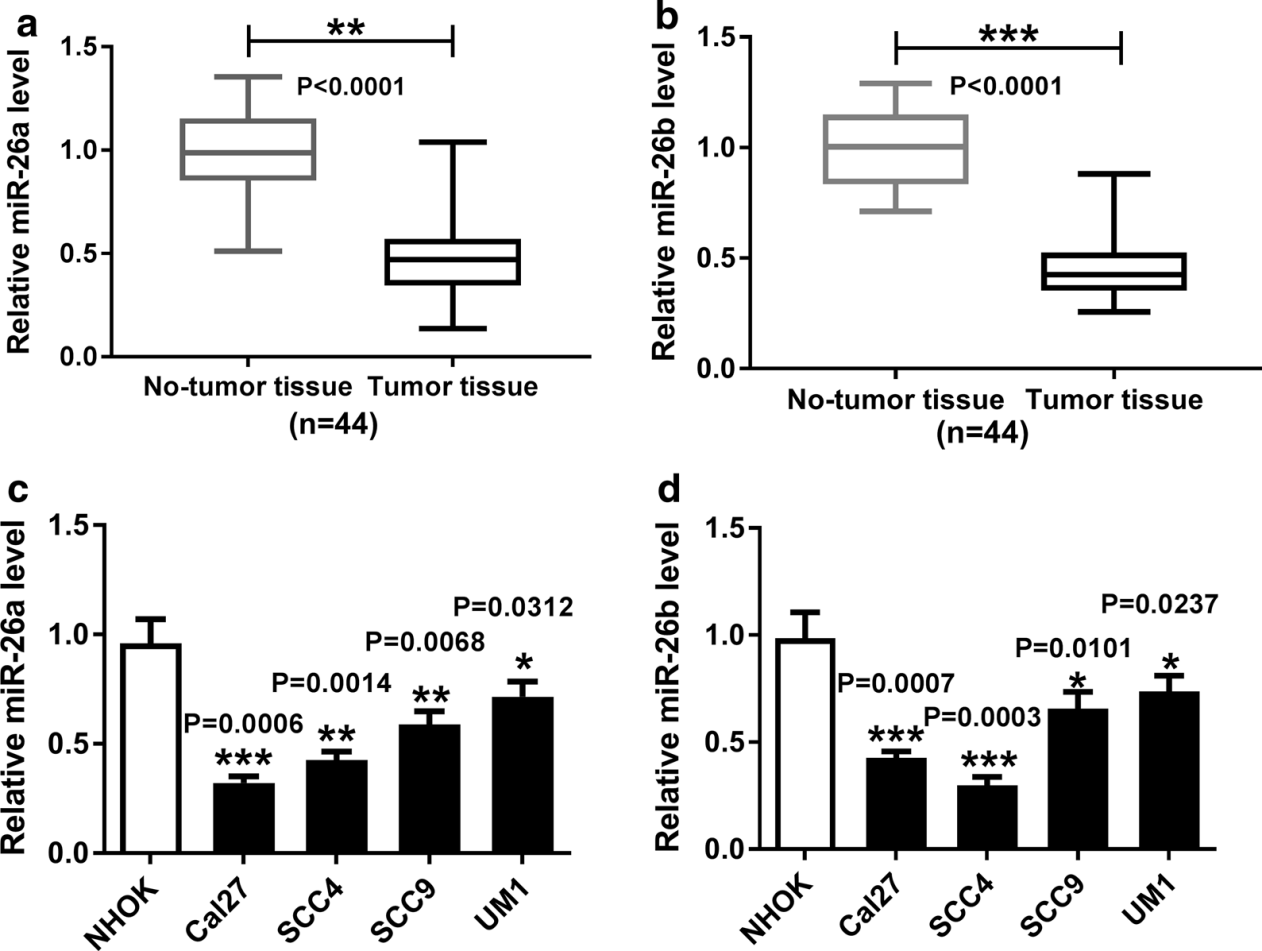
Both miR-26a and miR-26b were downregulated in TSCC tissues and cell lines. **a**, **b** RT-qPCR assay for the expression of miR-26a and miR-26b in TSCC tissues and adjacent normal tissues, n = 44. Statistical difference was analyzed by Wilcoxon signed-rank test. **c**, **d** RT-qPCR assay for the expression of miR-26a and miR-26b in NHOK cells and four TSCC cell lines. *P < 0.05, **P < 0.01, ***P < 0.001, as determined by ANOVA analysis followed by Tukey test

The expression levels of miR-26a and miR-26b in 44 pairs of TSCC tissues (tumor tissue) and adjacent normal tissues (No-tumor tissue) were initially detected using RT-qPCR. We found that both miR-26a and miR-26b expression were significantly decreased in TSCC tissues, when compared with normal tissues (Fig. 1a, b. *P *< 0.0001; *P *< 0.0001), in concordance with the analysis result utilizing YM500v and starbase 3.0 (Additional file 5). Moreover, we also examined the expression of miR-26a and miR-26b in TSCC cell lines (Cal27, SCC4, SCC9 and UM1) and NHOK. As compared with NHOK cells, the four cell lines all showed apparently reduced expression of miR-26a and miR-26b (Fig. 1c, d. *P *= 0.0006, *P *= 0.0014, *P *= 0.0068, *P *= 0.0312; *P *= 0.0007, *P *= 0.0003, *P *= 0.0101, *P *= 0.00237).

### Overexpressed miR-26a and miR-26b repressed TSCC cell cycle, migration and invasion

**Fig. 2:**
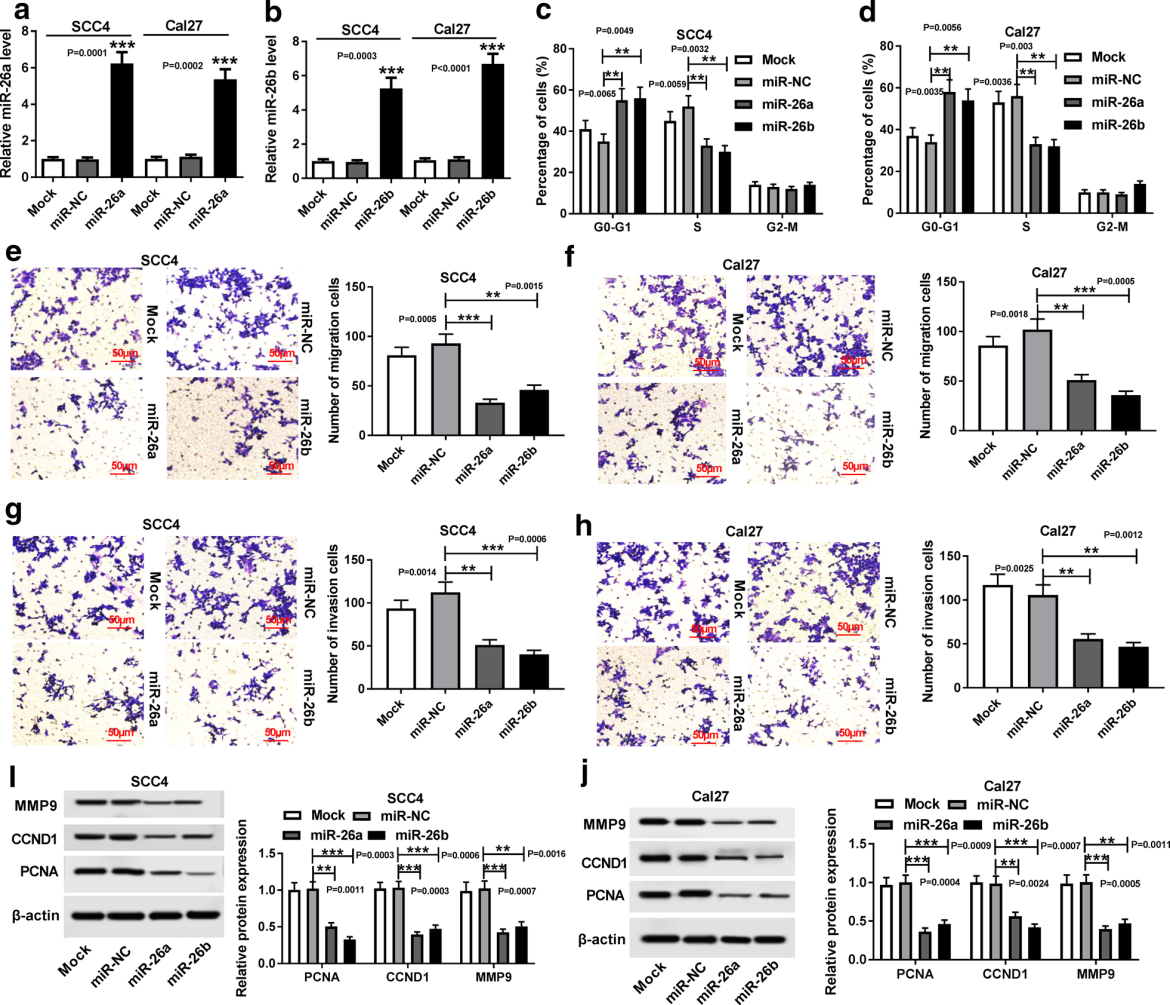
Overexpressed miR-26a and miR-26b repressed TSCC cell cycle, migration and invasion. SCC4 and Cal27 cells were transfected with Mock (blank control), miR-NC, miR-26a mimic or miR-26b mimic, respectively. **a**, **b** RT-qPCR assay for the expression of miR-26a and miR-26b in transfected SCC4 and Cal27 cells, as determined by ANOVA analysis followed by Tukey test. **c**, **d** Flow cytometry assay for the cell cycle of transfected SCC4 and Cal27 cells, as determined by ANOVA analysis followed by Tukey test. **e**–**h** Transwell assay for the migrated and invasive abilities of transfected SCC4 and Cal27 cells, as determined by ANOVA analysis followed by Tukey test. **i**–**j** Western blot assay for the protein levels of MMP9, CCND1 and PCNA in transfected SCC4 and Cal27 cells, as determined by ANOVA analysis followed by Tukey test. **P < 0.01, ***P < 0.001

To clarify the function of miR-26a and miR-26b in TSCC progression, SCC4 and Cal27 cells with miR-26a and miR-26b overexpression were constructed by transfection with miR-26a mimic or miR-26b mimic, respectively. Following RT-qPCR assay was employed to confirm the transfection efficiency and witnessed an about fivefold increasement of the expression of miR-26a/miR-26b, revealing that both miR-26a and miR-26b expression were highly enriched in transfected SCC4 and Cal27 cells (Fig. 2a, b. *P *= 0.0001, *P *= 0.0002; *P *= 0.0003, *P *< 0.0001). Flow cytometry assay showed that overexpression of miR-26a and miR-26b repressed the cell cycle of treated SCC4 and Cal27 cells, causing almost half reduction (Fig. 2c, d. *P *= 0.0065, *P *= 0.0049, *P *= 0.0059, *P *= 0.0032; *P *= 0.0035, *P *= 0.0056, *P *= 0.0036, *P *= 0.003). Moreover, Transwell assay indicated that the migrated and invasive abilities of miR-26a/miR-26b-overexpressed TSCC cells were obviously decreased when compared with the cells transfected with miR-NC (Fig. 2e–h. *P *= 0.0005, *P *= 0.0015; *P *= 0.0018, *P *= 0.0005; *P *= 0.0014, *P *= 0.0006; *P *= 0.0025, *P *= 0.0012). Following western blot analysis also revealed that upregulation of miR-26a/miR-26b could repress cell metastasis and cell cycle (Fig. 2i–j. *P *= 0.0011, *P *= 0.0003, *P *= 0.0003, *P *= 0.0006, *P *= 0.0007, *P *= 0.0016; *P *= 0.0004, *P *= 0.0009, *P *= 0.0024, *P *= 0.0007, *P *= 0.0005, *P *= 0.0011).

### Overexpression of miR-26a and miR-26b enhanced TSCC cell apoptosis, but repressed glycolysis

**Fig. 3:**
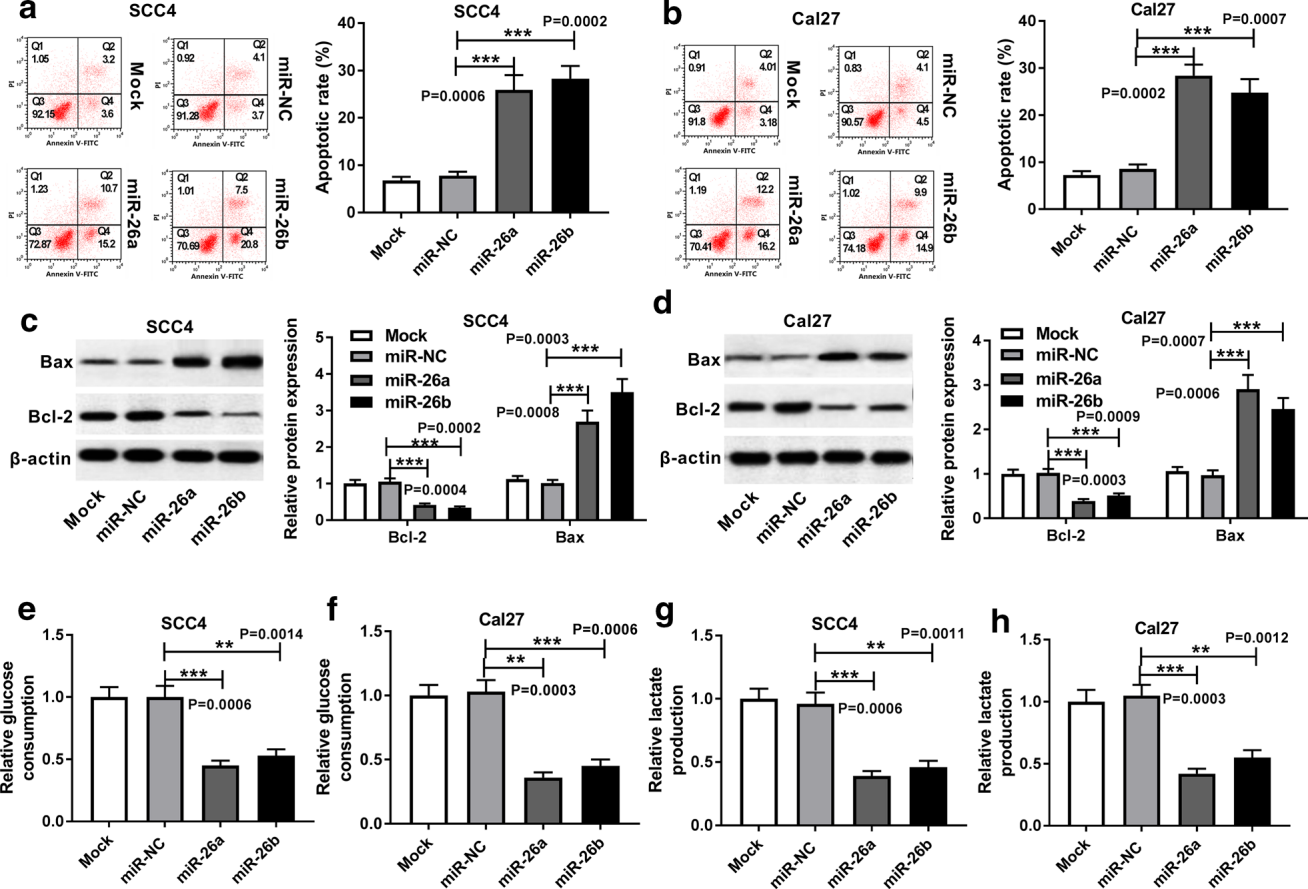
Overexpression of miR-26a and miR-26b enhanced TSCC cell apoptosis, but repressed glycolysis. SCC4 and Cal27 cells were transfected with Mock (blank control), miR-NC, miR-26a mimic or miR-26b mimic, respectively. **a**, **b** Flow cytometry assay for the apoptotic rate (Q2 + Q4) of transfected cells, as determined by ANOVA analysis followed by Tukey test. **c**, **d** Western blot assay for the protein levels of Bax and Bcl-2 in transfected SCC4 and Cal27 cells, as determined by ANOVA analysis followed by Tukey test. **e**–**f** Analysis for the glucose consumption of treated SCC4 and Cal27 cells, as determined by ANOVA analysis followed by Tukey test. **g**, **h** Analysis for the lactate production in transfected SCC4 and Cal27 cells was analyzed, as determined by ANOVA analysis followed by Tukey test. **P < 0.01, ***P < 0.001

To investigate the effect of miR-26a and miR-26b on cell apoptosis of TSCC cells, flow cytometry analysis was conducted. As exhibited in Fig. 3a, b, gain of both miR-26a and miR-26b distinctly elevated the apoptotic rate of transfected SCC4 and Cal27 cells, leading to about threefold augment (*P *= 0.0006, *P *= 0.0002; *P *= 0.0002, *P *= 0.0007). In addition, western blot assay was applied to clarify the effect of miR-26a and miR-26b on the protein levels of two apoptosis related proteins, Bax and Bcl-2, in treated cells. The results indicated that upregulated miR-26a and miR-26b conspicuously elevated Bax expression, while reduced Bcl-2 expression in transfected SCC4 and Cal27 cells (Fig. 3c, d. *P *= 0.0004, *P *= 0.0002, *P *= 0.0008, *P *= 0.0003; *P *= 0.0003, *P *= 0.0009, *P *= 0.0008, *P *= 0.0007). In addition, both the glucose consumption and lactate production in SCC4 and Cal27 cells with enriched miR-26a and miR-26b expression were lower than that in cells transfected miR-NC, about a half (Fig. 3e–h. *P *= 0.0006, *P *= 0.0014; *P *= 0.0003, *P *= 0.0006; *P *= 0.0006, *P *= 0.0011, *P *= 0.0003, *P *= 0.0012).

### Both miR-26a and miR-26b targeted PAK1

**Fig. 4:**
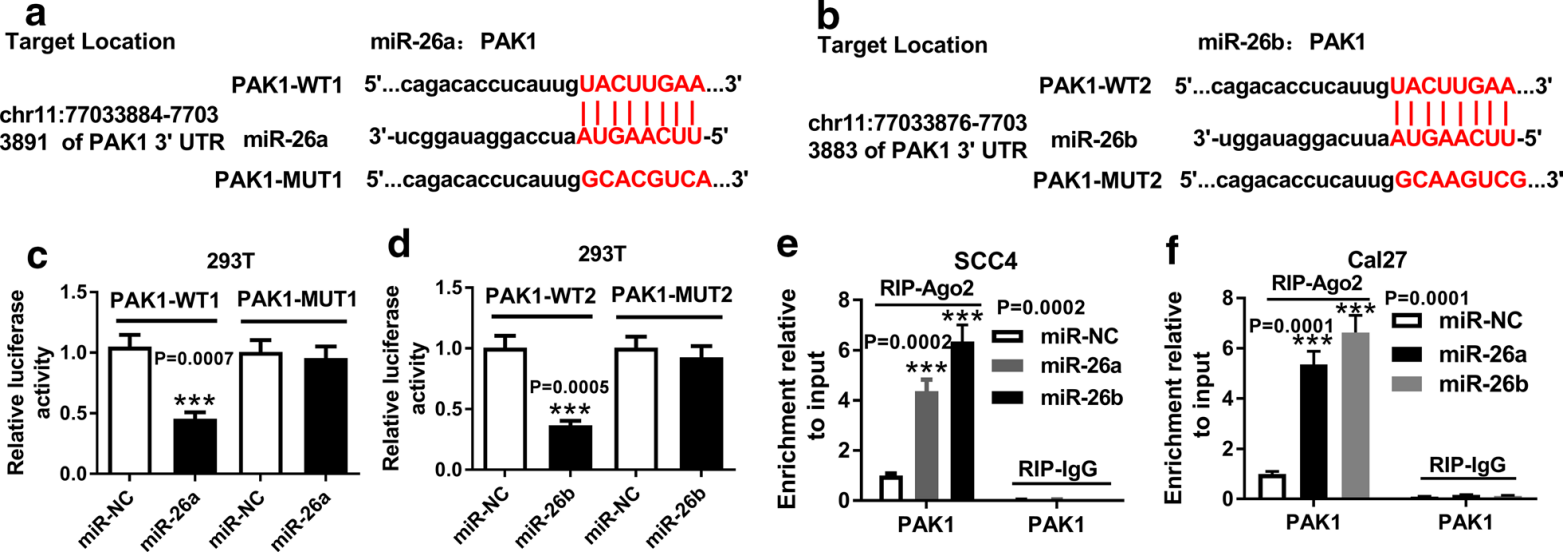
Both miR-26a and miR-26b targeted PAK1. **a**, **b** Predicted binding sites between miR-26a/miR-26b and PAK1 together with the mutant sites in PAK1-MUT. **c**, **d** Dual-luciferase reporter assay for the luciferase activity of 293T cells co-transfected with PAK1-WT or PAK1-MUT luciferase reporter and miR-26a mimic, miR-26b mimic or miR-NC at 48 h after transfection, as determined by ANOVA analysis followed by Tukey test. **e**, **f** RIP and RT-qPCR assays for the PAK1 enrichment level in IgG or Ago2 immunoprecipitation complex of SCC4 and Cal27 cells transfected with miR-26a mimic, miR-26b mimic or miR-NC after 48 h, as determined by ANOVA analysis followed by Tukey test. ***P < 0.001

To explore the underlying mechanism of miR-26a and miR-26b in TSCC cells, bioinformatics analysis was employed to seek out the potential target mRNAs of miR-26a and miR-26b, and indicated that PAK1 contained two common potential binding sites for miR-26a and miR-26b (Fig. 4a, b). To validate the prediction, we transfected 293T cells with PAK1-WT or PAK1-MUT, together with miR-26a or miR-26b. The dual-luciferase reporter assay suggested that overexpression of both miR-26a and miR-26b caused a notable reduction of luciferase activity (approximately 60%) in 293T cells treated with PAK1-WT1 or PAK1-WT2, but not in those treated with PAK1-MUT1 or PAK1-MUT2 (Fig. 4c, d. *P *= 0.0007, *P *= 0.0005). To further confirm the predicted interaction between miR-26a/miR-26b and PAK1, RIP assay was also performed. As shown in Fig. 4e, f, upregulation of miR-26a or miR-26b triggered the copious enrichment of PAK1 in Ago2 immunoprecipitation complex, suggesting the target interaction between miR-26a/miR-26b and PAK1 (*P *= 0.0002, *P *= 0.0002; *P *= 0.0001, *P *= 0.0001).

### PAK1 was highly expressed in TSCC tissues and cell lines, and was negatively regulated by miR-26a/miR-26b

**Fig. 5:**
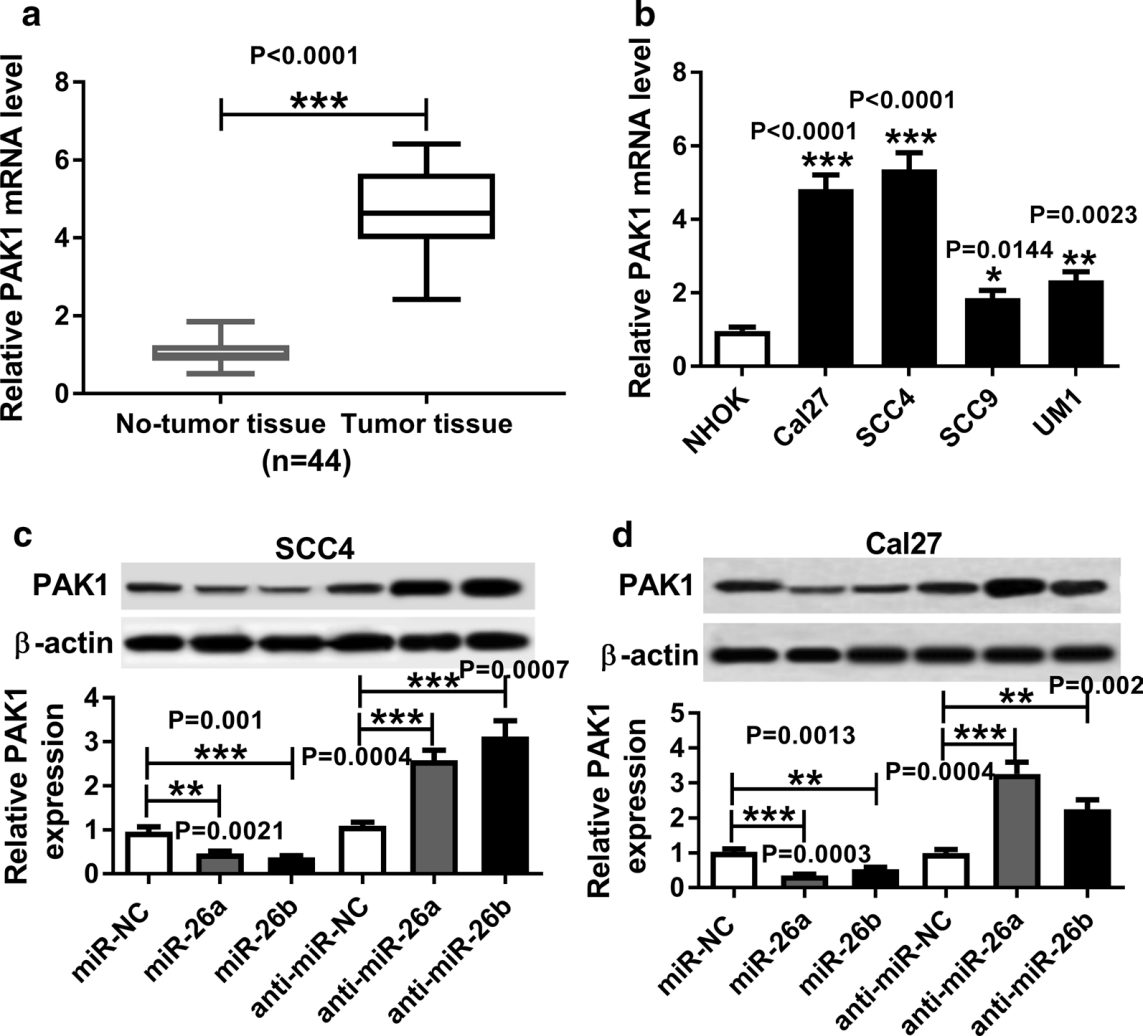
PAK1 was highly expressed in TSCC tissues and cell lines and was negatively regulated by miR-26a/miR-26b. **a** RT-qPCR assay for the expression of PAK1 in TSCC tissues and adjacent normal tissues, n = 44. Statistical difference was analyzed by Wilcoxon signed-rank test. **b** RT-qPCR assay for the PAK1 expression in NHOK cells and four TSCC cell lines, as determined by ANOVA analysis followed by Tukey test. **c**, **d** Western blot assay for PAK1 expression in SCC4 and Cal27 cells treated with miR-NC, miR-26a mimic, miR-26b mimic, anti-miR-NC, anti-miR-26a or anti-miR-26b, as determined by ANOVA analysis followed by Tukey test. *P < 0.05, **P < 0.01, ***P < 0.001

RT-qPCR assay demonstrated that PAK1 expression was notably elevated in 44 TSCC tissues and 4 TSCC cell lines compared with corresponding controls (Fig. 5a, b. *P *< 0.0001; *P *< 0.0001, *P *< 0.0001, *P *= 0.0144, *P *= 0.0023). SCC4 and Cal27 cells with miR-26a/miR-26b overexpression or inhibition were constructed to analyze the effect of miR-26a/miR-26b on PAK1 expression. Western blot assay revealed that upregulation of miR-26a/miR-26b evidently inhibited the protein level of PAK1, while downregulation of miR-26a/miR-26b induced opposite effect (Fig. 5c, d. *P *= 0.0021, *P *= 0.001, *P *= 0.0004, *P *= 0.0007; *P *= 0.0003, *P *= 0.0013, *P *= 0.0004, *P *= 0.002).

### Introduction of PAK1 partially reversed overexpressed miR-26a/miR-26b-mediated suppression of cell cycle, migration and invasion in TSCC cells

**Fig. 6:**
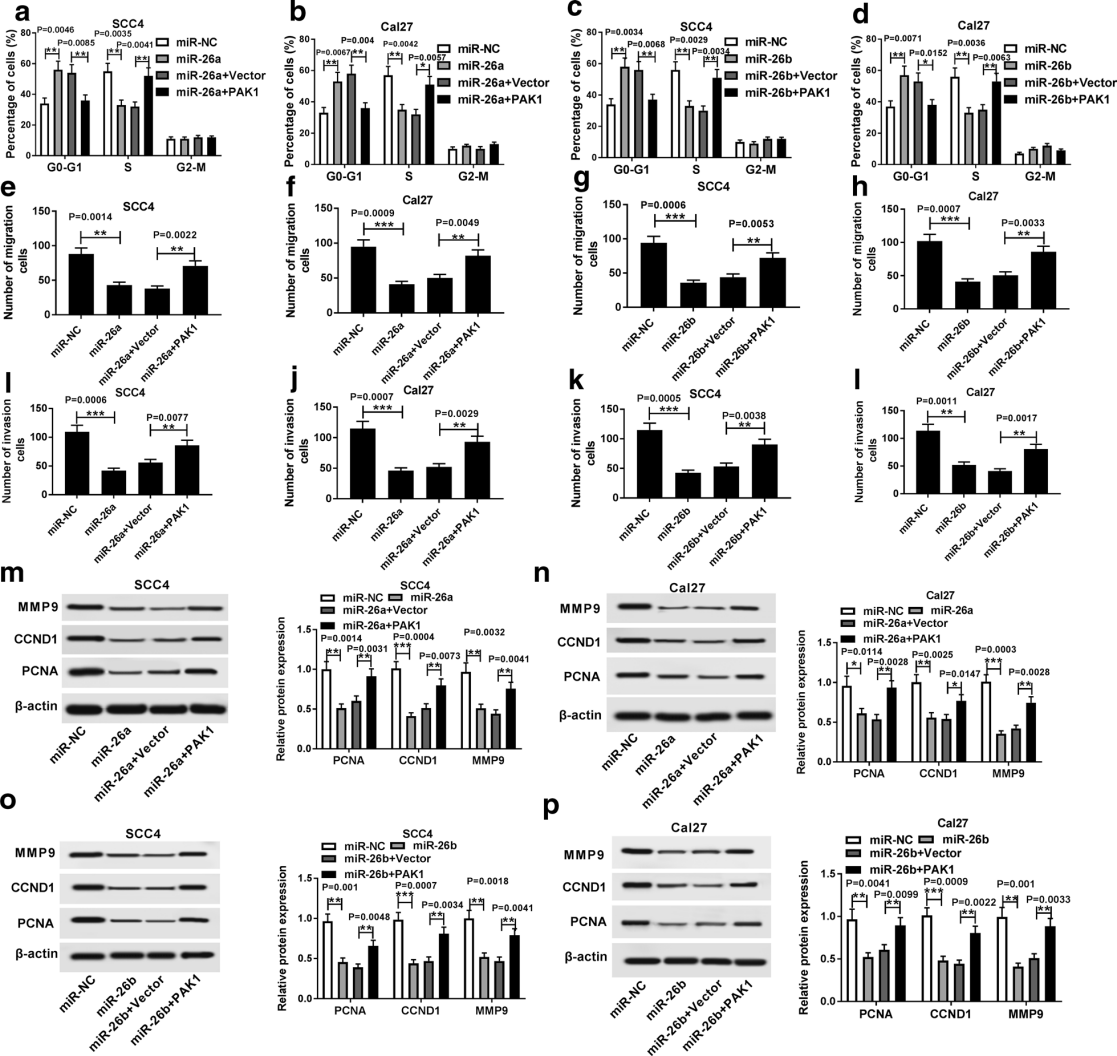
Introduction of PAK1 partially reversed overexpressed miR-26a/miR-26b-mediated suppression of cell cycle, migration and invasion in TSCC cells. SCC4 and Cal27 cells were transfected with miR-NC, miR-26a/miR-26b mimic, miR-26a/miR-26b mimic + pcDNA Vector or miR-26a/miR-26b mimic + pcDNA-PAK1. **a**–**d** Flow cytometry assay for the cell cycle of treated SCC4 and Cal27 cells, as determined by ANOVA analysis followed by Tukey test. **e**–**l** Transwell assay for the migrated or invasive abilities of SCC4 and Cal27 cells, as determined by ANOVA analysis followed by Tukey test. **m**–**p** Western blot assay for the protein levels of MMP9, CCND1 and PCNA in transfected SCC4 and Cal27 cells, as determined by ANOVA analysis followed by Tukey test. *P < 0.05, **P < 0.01, ***P < 0.001

To explore the regulatory effects of PAK1 upon the functional roles of miR-26a/miR-26b in TSCC progression, rescue experiments were performed in SCC4 and Cal27 cells by transfecting with miR-NC, miR-26a/miR-26b mimic, miR-26a/miR-26b mimic + pcDNA Vector or miR-26a/miR-26b mimic + pcDNA-PAK1. Flow cytometry analysis proved that SCC4 and Cal27 cells transfected with miR-26a/miR-26b mimic showed an evident decline of the cell cycle compared with miR-NC-transfected cells, which was effectively overturned by upregulated PAK1 (Fig. 6a–d. *P *= 0.0046, *P *= 0.0085, *P *= 0.0035, *P *= 0.0041; *P *= 0.0067, *P *= 0.004, *P *= 0.0042, *P *= 0.0057; *P *= 0.0034, *P *= 0.0068, *P *= 0.0029, *P *= 0.0034; *P *= 0.0071, *P *= 0.0152, *P *= 0.0036, *P *= 0.0063). Transwell assay revealed that gain of both miR-26a and miR-26b remarkably repressed the migrated and invasive abilities of SCC4 and Cal27 cells, while accompanied upregulation of PAK1 conspicuously abolished the repressive effect of the migrated and invasive abilities of SCC4 and Cal27 cells induced by miR-26a and miR-26b (Fig. 6e–l. *P *= 0.0014, *P *= 0.0022; *P *= 0.0009, *P *= 0.0049; *P *= 0.0006, *P *= 0.0053; *P *= 0.0007, *P *= 0.0033; *P *= 0.0006, *P *= 0.0077; *P *= 0.0007, *P *= 0.0029; *P *= 0.0005, *P *= 0.0038; *P *= 0.0011, *P *= 0.0017). As shown in Fig. 6m–p, western blot analysis suggested that introduction of PAK1 almost reversed the miR-26a/miR-26b-induced the reduction in the protein levels of MMP9, CCND1 and PCNA in SCC4 and Cal27 cells (*P *= 0.0014, *P *= 0.0031, *P *= 0.0004, *P *= 0.0073, *P *= 0.0032, *P *= 0.0041; *P *= 0.0114, *P *= 0.0028, *P *= 0.0025, *P *= 0.0147, *P *= 0.0003, *P *= 0.0028; *P *= 0.001, *P *= 0.0048, *P *= 0.0007, *P *= 0.0034, *P *= 0.0018, *P *= 0.0041; *P *= 0.0041, *P *= 0.0099, *P *= 0.0009, *P *= 0.0022, *P *= 0.001, *P *= 0.0033). Therefore, these data demonstrated that introduction of PAK1 partially reverted the suppression of cell cycle, migration and invasion in TSCC cells induced by miR-26a and miR-26b. In other words, miR-26a/miR-26b repressed TSCC proliferation and metastasis by downregulating PAK1.

### Upregulation of PAK1 weakened promotion of apoptosis and inhibition of glycolysis induced by miR-26a and miR-26b in TSCC cells

**Fig. 7:**
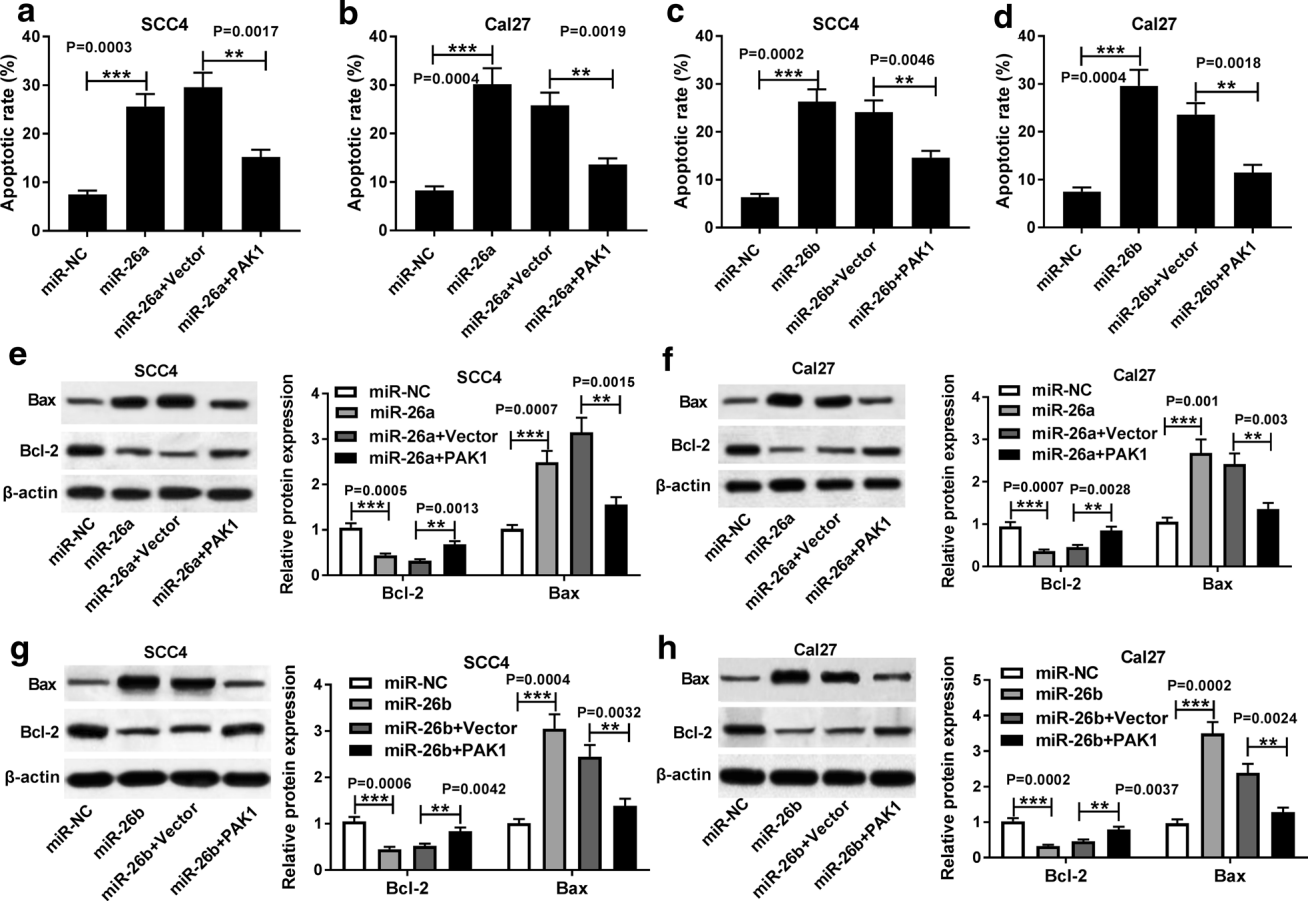
Upregulation of PAK1 weakened promotion of apoptosis induced by miR-26a and miR-26b in TSCC cells. SCC4 and Cal27 cells were transfected with miR-NC, miR-26a/miR-26b mimic, miR-26a/miR-26b mimic + pcDNA Vector or miR-26a/miR-26b mimic + pcDNA-PAK1. **a**–**d** Flow cytometry assay for the apoptotic rate (Q2 + Q4) of transfected SCC4 and Cal27 cells, as determined by ANOVA analysis followed by Tukey test. **e**–**h** Western blot assay for the protein levels of Bax and Bcl-2 in treated SCC4 and Cal27 cells, as determined by ANOVA analysis followed by Tukey test. **P < 0.01, ***P < 0.001

**Fig. 8:**
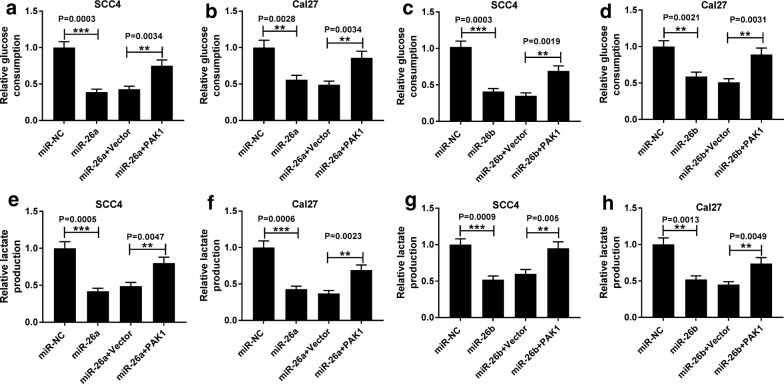
Upregulated PAK1 attenuated inhibition of glycolysis caused by miR-26a and miR-26b in TSCC cells. SCC4 and Cal27 cells were transfected with miR-NC, miR-26a/miR-26b mimic, miR-26a/miR-26b mimic + pcDNA Vector or miR-26a/miR-26b mimic + pcDNA-PAK1. **a**–**d** Analysis for the glucose consumption of treated SCC4 and Cal27 was assessed by commercial kits, as determined by ANOVA analysis followed by Tukey test. **e**–**h** Analysis for the lactate production in transfected SCC4 and Cal27 was evaluated, as determined by ANOVA analysis followed by Tukey test. *P < 0.05, **P < 0.01, ***P < 0.001

Next, we performed rescue experiments to clarify whether PAK1 could reverse the augment of apoptosis and reduction of glycolysis in TSCC cells triggered by miR-26a/miR-26b. Flow cytometry analysis manifested that upregulated miR-26a and miR-26b contributed to cell apoptosis of SCC4 and Cal27 cells, while reintroduction of PAK1 partially abrogated the impact of miR-26a/miR-26b (Fig. 7a–d. *P *= 0.0003, *P *= 0.0017; *P *= 0.0004, *P *= 0.0019; *P *= 0.0002, *P *= 0.0046; *P *= 0.0004, *P *= 0.0018). In addition, increased expression of miR-26a and miR-26b elevated Bax expression but blocked Bcl-2 expression, which was significantly reversed by co-transfection with pcDNA-PAK1 (Fig. 7e–h. *P *= 0.0005, *P *= 0.0013, *P *= 0.0007, *P *= 0.0015; *P *= 0.0007, *P *= 0.0028, *P *= 0.001, *P *= 0.003; *P *= 0.0006, *P *= 0.0042, *P *= 0.0004, *P *= 0.0032; *P *= 0.0002, *P *= 0.0037, *P *= 0.0002, *P *= 0.0024). As for glycolysis, gain of miR-26a/miR-26b effectively retarded glucose consumption and lactate production of SCC4 and Cal27 cells, but simultaneous introduction of PAK1 abated the inhibitory influence (Fig. 8a–h. *P *= 0.0003, *P *= 0.0034; *P *= 0.0028, *P *= 0.0034; *P *= 0.0003, *P *= 0.0019; *P *= 0.0021, *P *= 0.0031; *P *= 0.0005, *P *= 0.0047; *P *= 0.0006, *P *= 0.0023; *P *= 0.0009, *P *= 0.005, *P *= 0.0013, *P *= 0.0049). Hence, above outcomes suggested that upregulated PAK1 overturned elevation of apoptosis and reduction of glycolysis induced by miR-26a and miR-26b in TSCC cells, indicating that miR-26a/miR-26b exerted promoted effect on cell apoptosis and inhibitory effect on glycolysis by targeting PAK1.

### Upregulation of miR-26a/miR-26b blocked TSCC tumor growth in vivo

**Fig. 9:**
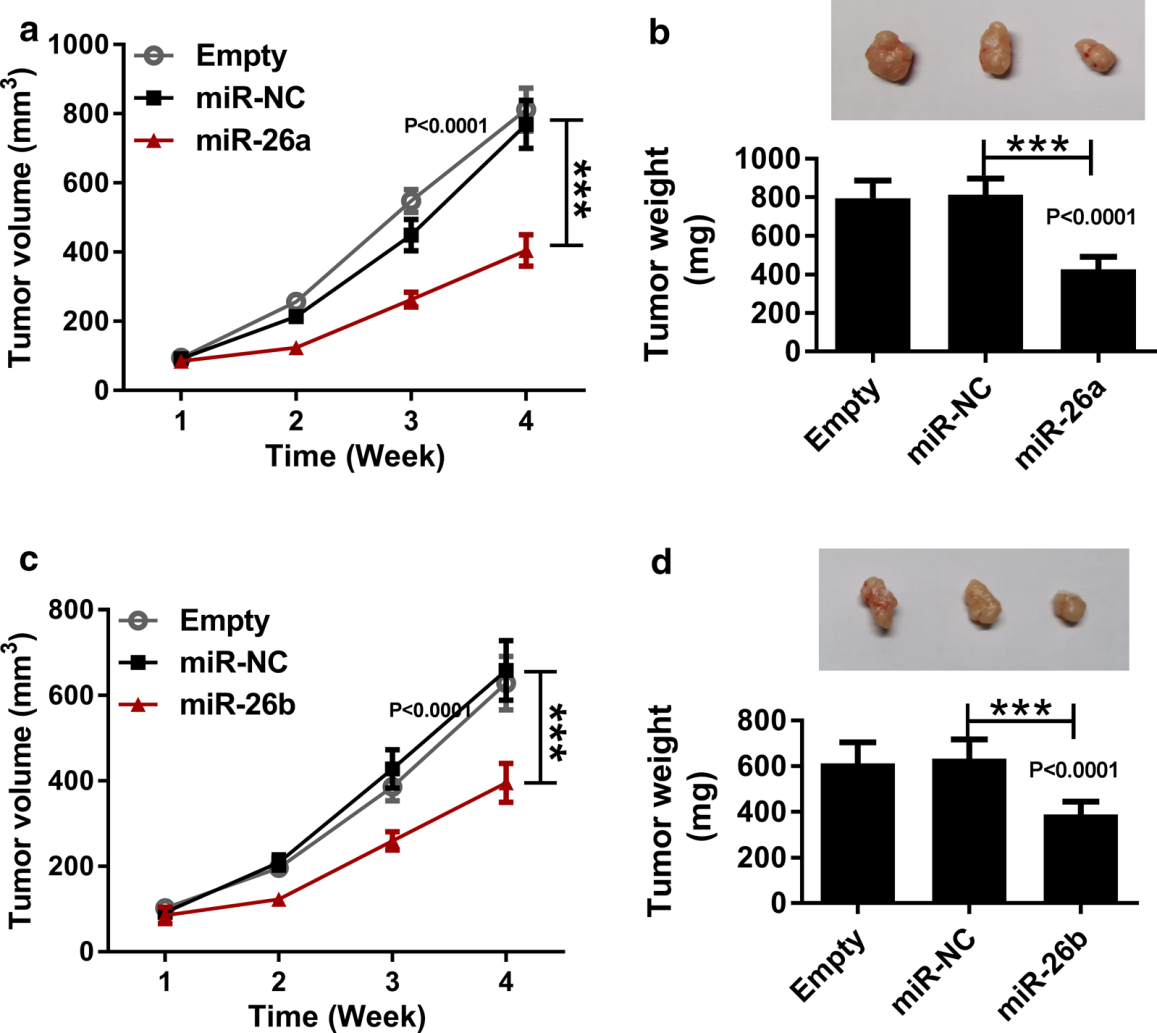
Upregulation of miR-26a/miR-26b blocked TSCC tumor growth in vivo. SCC4 cells stably expressing miR-NC, miR-26a and miR-26b were injected into nude mice (n = 5). **a**, **c** The volume of generated tumors, as determined by ANOVA analysis followed by Tukey test. **b**, **d** The weight of generated tumors, as determined by ANOVA analysis followed by Tukey test. ***P < 0.001

Later, we evaluated the function roles of miR-26a/miR-26b, we established nude mice model via injecting SCC4 cells stably expressing miR-NC, miR-26a and miR-26b. We found that miR-26a/miR-26b overexpression triggered approximately 45% reduction of the volume and weight of formed tumor relative to mice in miR-NC group (Fig. 9a–d. *P *< 0.0001; *P *< 0.0001; *P *< 0.0001; *P *< 0.0001).

## Discussion

In the current study, we observed low-level expression of miR-26a/miR-26b in TSCC tumor tissues and four TSCC cell lines. Overexpressed miR-26a/miR-26b inhibited cell cycle, migration, invasion and glycolysis but promoted apoptosis of SCC4 and Cal27 cells in vitro and hindered tumor growth in vivo. PAK1 was conformed as the common target of miR-26a and miR-26b. Furthermore, rescue experiments suggested that miR-26a/miR-26b exerted their tumor-suppression functions via targeting PAK1.

Emerging evidence have confirmed the important function of microRNAs in cancer development and tumorigenesis, including TSCC, acting as potent oncogenes or tumor suppressors [19]. Dysregulated microRNAs are also involved in the diagnosis and cure of TSCC [20]. In our study, we validated that miR-26a/miR-26b was downregulated in TSCC tumor tissues and cells, which was similar to former reports [11, 12]. The downregulation of miR-26a has been reported in various human malignancies, such as osteosarcoma (OS) [21], bladder cancer [22], breast cancer [23]. Liu and his colleagues revealed that miR-26a abundance was frequently decreased in OS tissues and cells, and miR-26a restrained the mobility of OS cells through targeting high-mobility group A1 (HMGA1) [21]. Zhao et al. found that miR-26a may be related to human breast carcinogenesis, which restrains tumor cell proliferation by targeting HMGA1 [23]. In addition, miR-26b was downregulated in locally advanced and inflammatory breast cancer [24] and non-small-cell lung cancer [25], functioning as tumor suppressor. To sum up, miR-26a/miR-26b might serve as tumor suppressed factors in TSCC.

TSCC cells with miR-26a/miR-26b overexpression were constructed through transient transfection to study the role of miR-26a/miR-26b in the celluar behaviors, including cell cycle, migration, invasion, apoptosis and glycolysis. Jia et al. pointed out that introduction of miR-26a caused the inhibition on cell proliferation, as well as promotion on cell apoptosis of TSCC cells (SCC-15 and CAL27 cells) [12]. Yang et al. demonstrated that miR-26a/miR-26b hampered colorectal cancer cell aggressiveness via regulating FUT4 expression [26]. A former study performed by Xu et al. indicated that overexpressed miR-26a effectively impeded multiple myeloma cell growth and delayed tumor growth in vivo through regulating CDK6 enrichment [27]. Moreover, miR-26b level was proved to be reduced in TSCC and hindered proliferation and metastasis in TSCC cells through targeting COX-2 [11]. Du and his partners disclosed that miR-26b exerted its tumor suppressive role through the modulation of glycolytic metabolism in OS cells, showing as regulating expression of glycolytic components, LDHA and GLUT-1 [28]. By analyzing pathway of variant expressed protein, miR-26b is the upstream of the glycolysis/TCA cycle and cytoskeletal modulation via Rho GTPases, implying its significance in the breast cancer progression [29]. Analogously, we manifested that introduction of miR-26a or miR-26b restrained cell cycle, migration, invasion and glycolysis, while facilitated apoptosis of SCC4 and Cal27 cells. Additionally, xenograft tumor assay uncovered the tumor suppressor role of miR-26a/miR-26b in vivo, causing smaller tumor size and weight.

Here, we took effort to explore other potential mechanism of miR-26a/miR-26b. Bioinformatic analysis, dual-luciferase reporter assay as well as RIP assay were conducted to seek the downstream gene of miR-26a/miR-26b, and identified PAK1 as a target. Also, PAK1 enrichment was upregulated in TSCC tumor tissues and cell lines in contrast to corresponding negative controls, as expected.

PAK1, a prototype of group I PAKs, was known to function in the key steps of human cancer progression; and its roles were well studied in multiple malignancies, including breast cancer, colon cancer, lung cancer, melanoma, prostate cancer, ovarian cancer and TSCC [30, 31]. For instance, PAK1 was demonstrated to be a target of miR-7 to participate in regulating the proliferation and metastasis of thyroid cancer cells [15]. Furthermore, PAK1, a target gene of miR-494, functioned as an oncogenic factor in breast cancer via activating MAPK signaling pathway and remodeling cytoskeletal [32]. In non-small cell lung cancer (NSCLC), PAK1 served as clinical biomarker and its level was elevated in NSCLC tissues and cells; also, overexpressed PAK1 reverted the inhibitory impact of miR-98 on growth and mobility of NSCLC cells [33]. Parvathy et al. claimed that PAK1 played a significant part in remodeling cytoskeletal, thus influencing the motility and aggressiveness of oral squamous cell carcinoma cells [30]. LncRNA MALAT1/miRNA-140-5p/PAK1 axis modulated the proliferative and mobility abilities of TSCC cells [18]. In our study, rescue experiments were performed and gain of PAK1 partially overturned the upregulation of miR-26a and miR-26b-mediated suppression of cell cycle, migration, invasion and glycolysis, as well as elevation of apoptosis, indicating that miR-26a/miR-26b regulated cell cycle, metastasis, apoptosis and glycolysis in TSCC cells via downregulating PAK1.

There still exist some limitations in our project. For example, based on the ceRNA hypothesis, circRNAs could repress expression levels of their target miRNAs, so as to alter the expression and function of mRNAs [34], which urging us to search the upstream circRNAs for better understanding of TSCC progression. Moreover, NF-κB pathway was recognized as an important signal pathway involved in the occurrence and development of various human tumors, including TSCC [35]. We would investigate the impact of miR-26a/miR-26b on the activation of this signal pathway.

## Conclusion

Taken together, our study proved that miR-26a/miR-26b was apparently downregulated, while PAK1 was highly expressed in TSCC tissues and cells. Moreover, gain of miR-26a or miR-26b blocked cell cycle, migration, invasion and glycolysis but promoted apoptosis by inhibiting PAK1 in vitro. Overexpression of miR-26a/miR-26b also inhibited TSCC tumor propagation in vivo. Above outcomes disclosed that miR-26a/miR-26b acted as tumor suppressor and might function as therapeutic target of TSCC patients.

## Supplementary information

**Additional file 1.** Full uncropped immunoblot images with molecular weight markers of Fig. 2i, j and 3c, d.

**Additional file 2.** Full uncropped immunoblot images with molecular weight markers of Fig. 5c, d.

**Additional file 3.** Full uncropped immunoblot images with molecular weight markers of Fig. 6m–p.

**Additional file 4.** Full uncropped immunoblot images with molecular weight markers of Fig. 7e-h.

**Additional file 5.**  Expression of miR-26a/miR-26b in head and neck squamous cell carcinomas. Analyzation of miR-26a/miR-26b expression in head and neck squamous cell carcinomas using YM500v (a, b) and starBase 3.0 (c, d).

## Data Availability

The analyzed data sets generated during the present study are available from the corresponding author on reasonable request.

## Abbreviations

TSCC: Tongue squamous cell carcinoma
PAK1: p21 Activated Kinase 1
RT-qPCR: Reverse transcription-quantitative polymerase chain reaction
RIP: RNA immunoprecipitation
MiRNAs: MicroRNAs
MRNAs: Message RNAs
NHOK: Normal human oral keratinocyte
DMEM: Dulbecco’s Modified Eagle Medium
FBS: Fetal bovine serum
GAPDH: Glyceraldehyde-3-phosphate dehydrogenase
MMP9: Matrix metalloproteinase 9
CCND1: Cyclin D1
PCNA: Proliferating Cell Nuclear Antigen
Bcl-2: B cell lymphoma-2
Bax: Bcl-2-Associated X
SDS-PAGE: Sodium dodecyl sulphate–polyacrylamide gel electrophoresis
OS: Osteosarcoma
HMGA1: High-mobility group A1
NSCLC: Non-small cell lung cancer
