# Supplementary figures and images for: MiR-26a/miR-26b represses tongue squamous cell carcinoma progression by targeting PAK1
Source: Cancer Cell Int. 2020 Mar 14;20:82. doi: 10.1186/s12935-020-1166-6 (PMC7071636; doi:10.1186/s12935-020-1166-6)

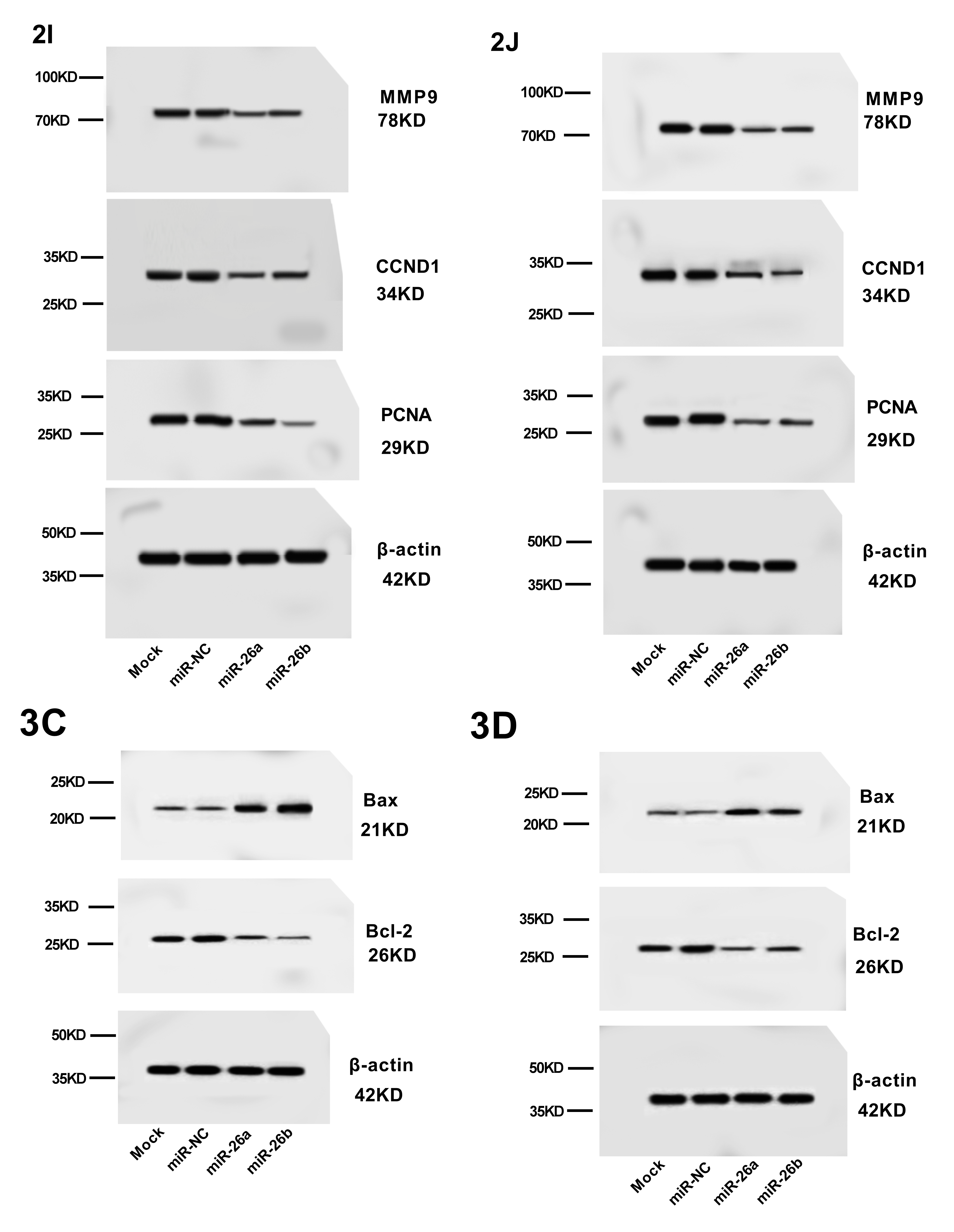

Supplement: Supplementary file 1 — Additional file 1. Full uncropped immunoblot images with molecular weight markers of Fig. 2i, j and 3c, d. [file 12935_2020_1166_MOESM1_ESM.tif]

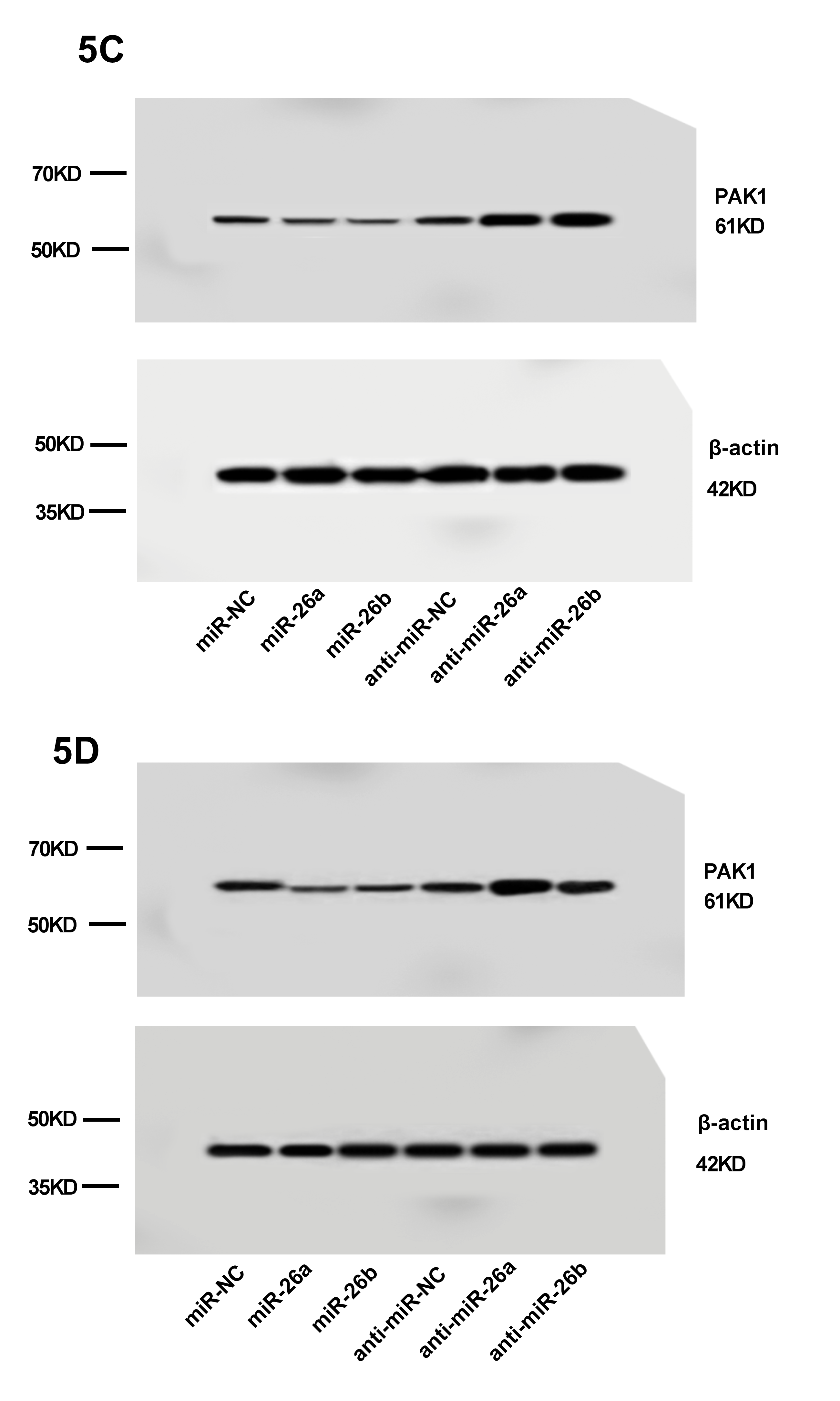

Supplement: Supplementary file 2 — Additional file 2. Full uncropped immunoblot images with molecular weight markers of Fig. 5c, d. [file 12935_2020_1166_MOESM2_ESM.tif]

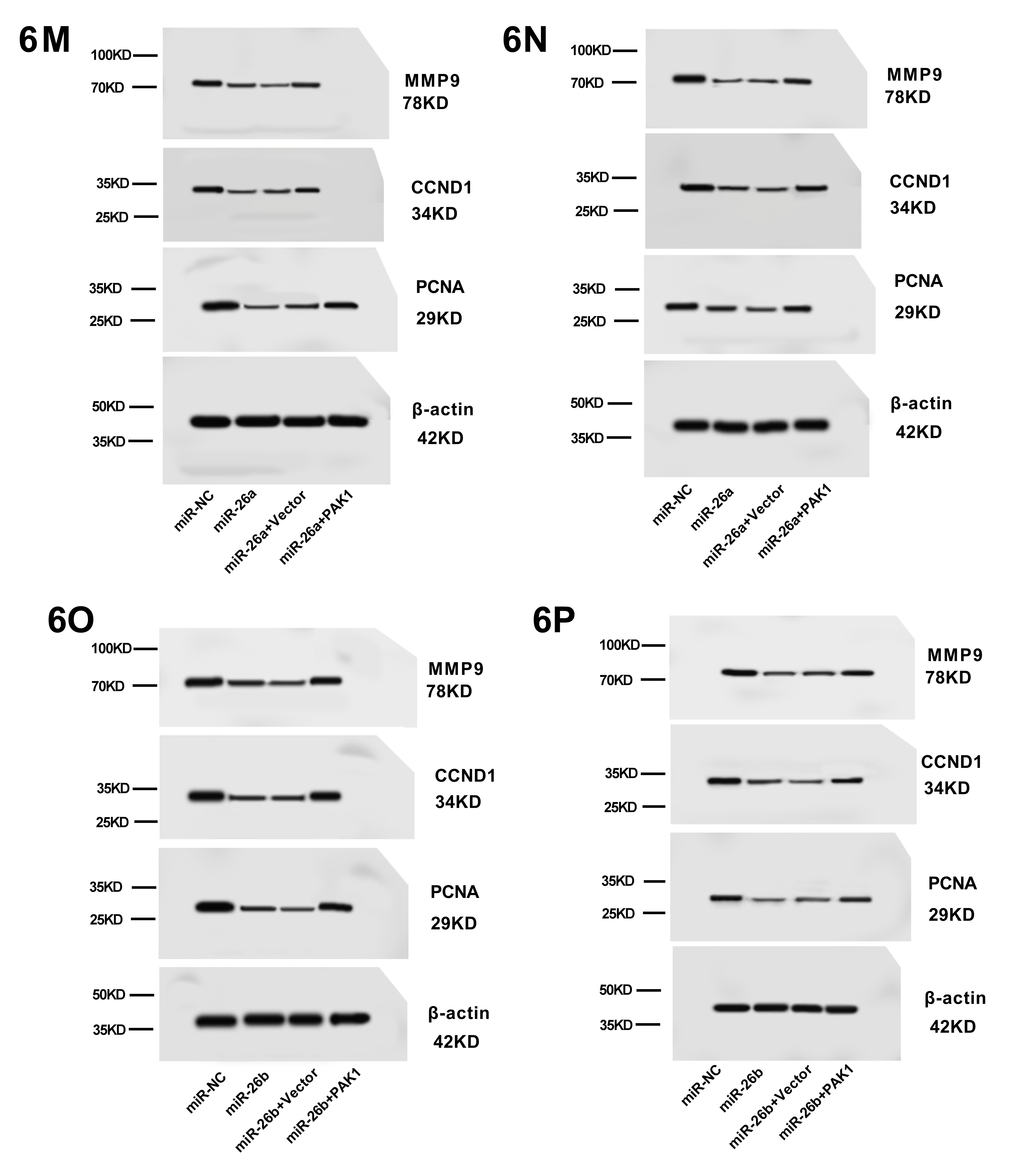

Supplement: Supplementary file 3 — Additional file 3. Full uncropped immunoblot images with molecular weight markers of Fig. 6m–p. [file 12935_2020_1166_MOESM3_ESM.tif]

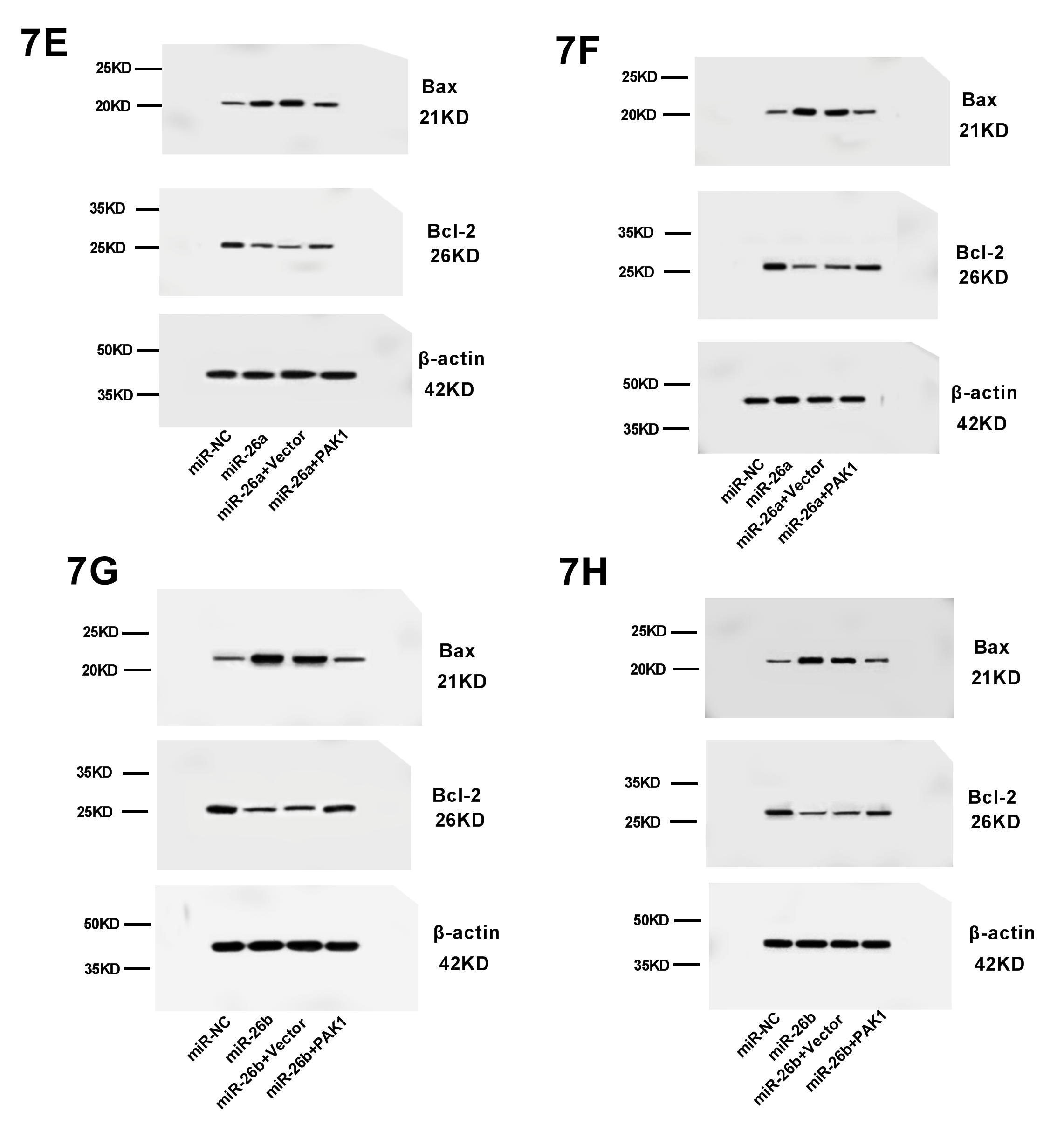

Supplement: Supplementary file 4 — Additional file 4. Full uncropped immunoblot images with molecular weight markers of Fig. 7e-h. [file 12935_2020_1166_MOESM4_ESM.tif]

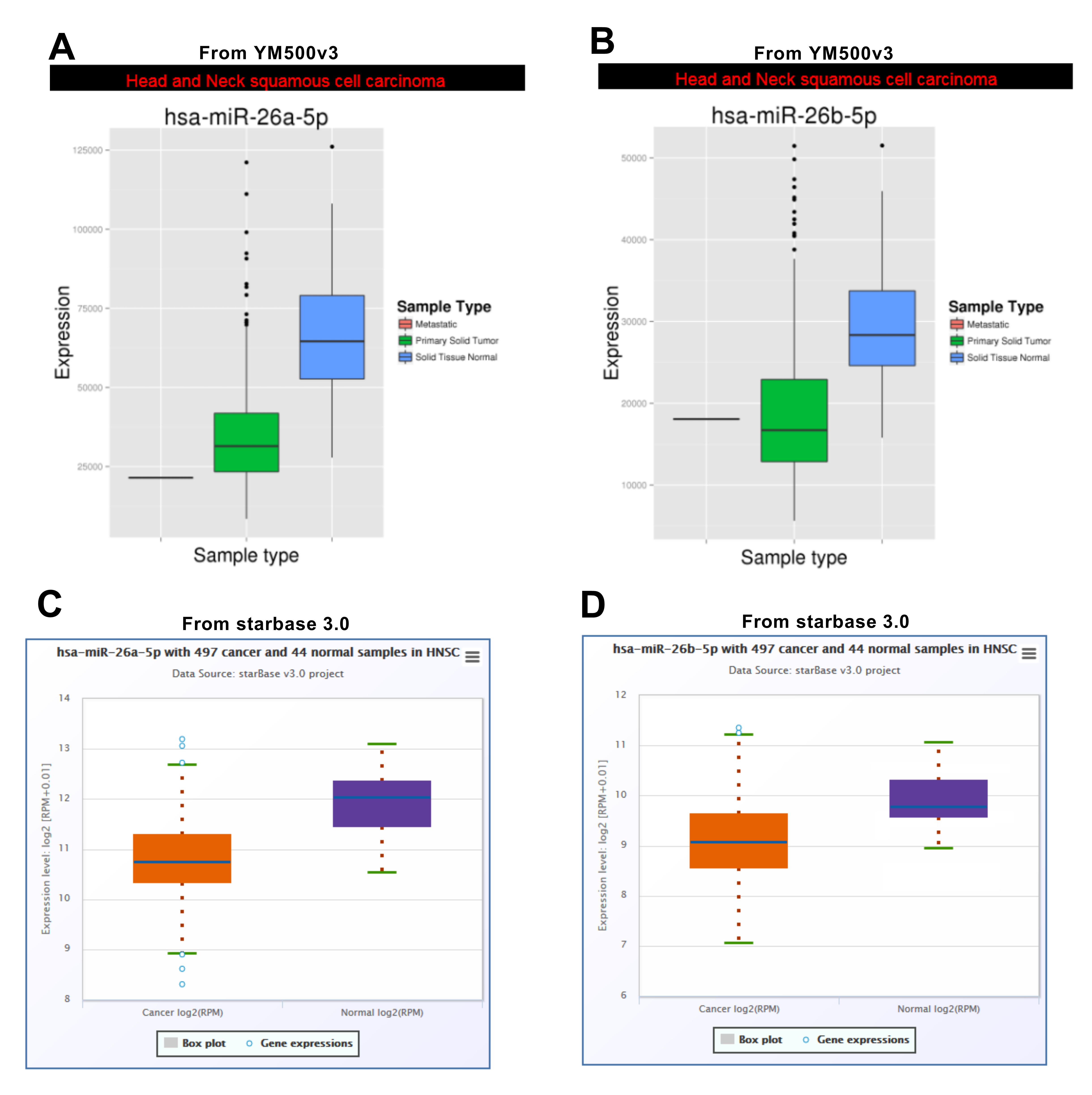

Supplement: Supplementary file 5 — Additional file 5. Expression of miR-26a/miR-26b in head and neck squamous cell carcinomas. Analyzation of miR-26a/miR-26b expression in head and neck squamous cell carcinomas using YM500v (a, b) and starBase 3.0 (c, d). [file 12935_2020_1166_MOESM5_ESM.tif]
